# A Highly Active Form of XCL1/Lymphotactin Functions as an Effective Adjuvant to Recruit Cross-Presenting Dendritic Cells for Induction of Effector and Memory CD8^+^ T Cells

**DOI:** 10.3389/fimmu.2018.02775

**Published:** 2018-11-27

**Authors:** Kazuhiko Matsuo, Kosuke Kitahata, Fumika Kawabata, Momo Kamei, Yuta Hara, Shiki Takamura, Naoki Oiso, Akira Kawada, Osamu Yoshie, Takashi Nakayama

**Affiliations:** ^1^Division of Chemotherapy, Kindai University Faculty of Pharmacy, Osaka, Japan; ^2^Laboratory of Cell Biology, Kindai University Faculty of Pharmacy, Osaka, Japan; ^3^Department of Immunology, Kindai University Faculty of Medicine, Osaka, Japan; ^4^Department of Dermatology, Kindai University Faculty of Medicine, Osaka, Japan; ^5^Kindai University, Osaka, Japan; ^6^The Health and Kampo Institute, Miyagi, Japan

**Keywords:** XCR1, XCL1, cross-presenting DC, CTL, adjuvant

## Abstract

The chemokine receptor XCR1 is known to be selectively expressed by cross-presenting dendritic cells (DCs), while its ligand XCL1/lymphotactin is mainly produced by activated CD8^+^ T cells and natural killer cells. Recent studies have shown that XCL1-antigen fusion proteins efficiently induce CD8^+^ T cell responses by preferentially delivering antigens to XCR1^+^ DCs. However, XCL1 *per se* was found to be a poor adjuvant for induction of CD8^+^ T cell responses. XCL1 is unique because of its lack of one of the two disulfide bonds commonly conserved in all other chemokines and thus has an unstable structure with a relatively weak chemokine activity. In the present study, we generated a variant form of murine XCL1 termed mXCL1-V21C/A59C that contained a second disulfide bond to stabilize its chemokine structure. We confirmed that mXCL1-V21C/A59C had much more potent chemotactic and calcium mobilization activities than the wild type XCL1 (mXCL1-WT). Intradermal injection of mXCL1-V21C/A59C, but not that of mXCL1-WT, significantly increased the accumulation of XCR1^+^CD103^+^ DCs in the injection site, and most of the accumulated XCR1^+^CD103^+^ DCs were found to take up co-injected ovalbumin (OVA). Furthermore, recruited XCR1^+^CD103^+^ DCs efficiently migrated to the draining lymph nodes and stayed for a prolonged period of time. Consequently, mXCL1-V21C/A59C strongly induced OVA-specific CD8^+^ T cells. The combination of OVA and mXCL1-V21C/A59C well protected mice from E.G7-OVA tumor growth in both prophylactic and therapeutic protocols. Finally, memory CTL responses were efficiently induced in mice immunized with OVA and mXCL1-V21C/A59C. Although intradermal injection of OVA and polyinosinic-polycytidylic acid (poly(I:C)) as an adjuvant also induced CD8^+^ T cell responses to OVA, poly (I:C) poorly recruited XCR1^+^CD103^+^ DCs in the injection site and failed to induce significant memory CTL responses to OVA. Collectively, our findings demonstrate that a highly active form of XCL1 is a promising vaccine adjuvant for cross-presenting DCs to induce antigen-specific effector and memory CD8^+^ T cells.

## Introduction

CD8^+^ cytotoxic T lymphocytes (CTLs) are the critical effector cells in adoptive cellular immunity to tumor cells and virus-infected cells. Dendritic cells (DCs), the professional antigen-presenting cells, process and present intracellular antigens such as viral and tumor antigens via the MHC class I pathway that induces antigen-specific CD8^+^ CTL responses. However, in the case of extracellular antigens such as subunit vaccines, DCs generally process and present antigens in the MHC class II pathway that preferentially activates CD4^+^ T cells. Consequently, current subunit vaccines tend to induce humoral immune responses rather than CD8^+^ CTL responses. To induce antigen-specific CTL responses, subunit vaccines need to be presented on the MHC class I in DCs through a process called cross-presentation. It is therefore of great importance to develop adjuvants that promote cross-presentation of extracellular antigens. A number of adjuvants have been studied in preclinical animal models, but very few meet the requirement of safety and efficacy for human use.

Mammalian DCs are now divided into several functional subsets, and specialized subsets are considered to be capable of cross-presentation (1). In mice, lymphoid tissue-resident CD8α^+^ DCs were originally reported to induce cross-presentation of extracellular antigens to naïve CD8^+^ T cells (2). In the mouse skin, CD103^+^CD11b^−^ DCs, but neither CD103^−^CD11b^+^ DCs nor epidermal Langerhans cells, were found to be the most potent subset for cross-presentation of antigens to naïve CD8^+^ T cells in draining lymph nodes (3). Of note, dermal CD103^+^CD11b^−^ DCs also express CD8α. CD141^+^ DCs in humans and CADM1^+^ DCs in macaques are now considered to be the functional homolog of murine CD103^+^ DCs (4). It is now known that the chemokine receptor XCR1 is selectively expressed by cross-presenting DCs in many mammalian species such as humans, mice, sheep, and swine, while its ligand XCL1/lymphotactin is mainly produced by activated CD8^+^ T cells and natural killer cells (2, 5, 6). Thus, the XCL1-XCR1 axis is likely to be specialized for cross-presentation.

Targeting antigens to cell surface molecules on DCs is an effective way to induce specific T cell responses. Surface molecules such as DEC205 and Clec9a have been explored as antigen delivery targets for cross-presenting DCs, but these molecules are also expressed by other immune cells (6). In contrast, XCR1 is highly selective for cross-presenting DCs in many mammalian species such as humans, macaques, sheep, and mice (6). Thus, several recent studies tested DNA vaccines encoding fusion molecules consisting of XCL1 and antigens to selectively deliver antigens to XCR1^+^ cross-presenting DCs and have demonstrated effective induction of CD8^+^ T cell responses (7–9). However, XCL1 *per se* used as an adjuvant for cross-presenting DCs failed to induce significant CD8^+^ T cell responses (9).

XCL1 is unique because it retains only one of the two disulfide bonds that are commonly conserved in all other chemokines. Thus, XCL1 has a relatively weak chemotactic activity, most probably because of its unstable structure (10). Indeed, Tuinstra et al. have shown that under physiological conditions, XCL1 exhibits a dynamic conformational equilibrium between two distinct structural species, the canonical chemokine form and another form which lacks XCR1 agonist activity (11). Tuinstra et al. have further shown that a variant form of human XCL1 termed XCL1-V21C/V59C which incorporated a second disulfide bond to stabilize the canonical chemokine form exhibited an enhanced chemotactic activity (12, 13). In the present study, based on the human XCL1-V21C/V59C, we generated the structurally stable form of murine XCL1 termed mXCL1-V21C/A59C and confirmed its potent chemotactic and calcium mobilization activities via XCR1. Furthermore, we demonstrated that intradermal injection of ovalbumin (OVA) with mXCL1-V21C/A59C as an adjuvant efficiently induced accumulation of XCR1^+^CD103^+^ DCs in the injection site and their migration to draining lymph nodes, resulting in a potent induction of effector and memory CD8^+^ T cell responses to OVA. Thus, we conclude that a stable form of XCL1 is a useful adjuvant for cross-presenting DCs.

## Materials and methods

### Mice

C57BL/6 mice at 7–10 weeks old were purchased from Japan SLC (Hamamatsu, Japan). OT-I mice, transgenic mice whose CD8^+^ T cells recognize the OVA257–264 (SIINFEKL) peptide in the context of H-2b on the C57BL/6 background, were kindly provided by Miyuki Azuma (Tokyo Medical and Dental University, Tokyo, Japan) with permission from William R. Heath (University of Melbourne, Victoria Australia) (14). Mice were maintained in specific pathogen-free conditions. All animal experiments in the present study were approved by the Center of Animal Experiments, Kindai University, and performed in accordance with the institutional guidelines.

### Cells

A mouse pre-B cell line L1.2 was kindly provided by Eugene Butcher (Stanford University School of Medicine, Stanford, CA). L1.2 cell lines stably expressing mouse chemokine receptors were generated using a retroviral vector pMX-IRES-EGFP as described previously (15). E.G7-OVA cells (OVA cDNA-transfectant of EL4 cells) were purchased from American Type Culture Collection (ATCC; Manassas, VA) and maintained in RPMI1640 medium supplemented with 10% FBS, 50 μM 2-ME, and 400 μg/ml G418. 293-F cells were purchased from Thermo Fisher Scientific Inc. (Waltham, MA) and maintained in Free Style 293 Expression Medium (Thermo Fisher Scientific).

### Cell isolation

Skin cells were isolated as described previously (16). In brief, skin tissues taken from mice were incubated for 60 min at 37°C in RPMI1640 supplemented with 0.24 mg/ml collagenase A (Roche; Basel, Switzerland) and 40 U/ml DNase I (Thermo Fisher Scientific). After shaking vigorously for 10 s, cell suspensions were filtered through a 70-μm cell strainer. Spleen cells were prepared by mashing spleens through a 70-μm cell strainer and lysing erythrocytes with ACK lysis buffer (150 mM NH_4_Cl, 10 mM KHCO_3_ and 0.1 mM Na_2_EDTA, pH 7.2).

### Production of mXCL1-WT and mXCL1-V21C/A59C

To generate the expression vectors for wild-type mXCL1 (mXCL1-WT) and its variant with two disulfide bonds (mXCL1-V21C/A59C), the cDNAs for mXCL1-WT and mXCL1-V21C/A59C containing NheI and NotI sites were chemically synthesized (Thermo Fisher Scientific). These cDNA fragments were digested with NheI and NotI and cloned into the expression vector pcDNA3.1 (Thermo Fisher Scientific). The transfection procedure was performed using FreeStyle MAX Reagent (Thermo Fisher Scientific). Lipid-DNA complexes were formed by mixing 37.5 μl FreeStyle MAX Reagent and 37.5 μg DNA per 30 ml culture. This complex was added to 30 ml media containing 293-F cells at 1 × 10^6^ cells/ml. Three days after transfection, the recombinant proteins were purified from culture supernatants using the His GraviTrap Kit (GE Healthcare Ltd., UK) following the manufacture's protocol. In brief, culture supernatants were pooled and applied to His GraviTrap 1-ml column equilibrated with 5 column volume of a binding buffer (20 mM sodium phosphate, 500 mM NaCl, and 20 mM imidazole, pH 7.4). After washing with 10 column volume of the binding buffer, the recombinant proteins were eluted by 3 column volume of an elution buffer (20 mM sodium phosphate, 500 mM NaCl, and 500 mM imidazole, pH 7.4). The fractions containing proteins were pooled and concentrated using Amicon Ultra filtration cartridges (3 kDa, Millipore Corporation, Billerica, MA). The protein concentration was determined by Protein Assay BCA Kit (Wako, Osaka, Japan).

### Immunoblot analysis

For immunoblot analysis, purified proteins were diluted in 5 × SDS buffer (225 mM Tris-HCl, 50% Glycerol, 5% SDS, 0.05% BPB, 250 mM DTT) and boiled for 5 min. Proteins were run by electrophoresis on an SDS-polyacrylamide gel (reduced; 15%) and electrophoretically transferred to a polyvinylidene fluoride membrane (Merck Millipore, Burlington, MA, USA). Membranes were blocked with 5% skim milk/TBS-T (20 mM Tris-HCl, 137 mM NaCl and 0.1% Tween 20, pH 7.6) and incubated with peroxidase conjugated-anti-6 × histidine monoclonal antibody (clone 9C11) (1:5,000; Wako Pure Chemical Industries, Osaka, Japan) in 5% skim milk/TBS-T for 2 h at room temperature. After washing, bands were visualized using Chemi-Lumi One Super (Nacalai Tesque, Kyoto, Japan) and analyzed on an ImageQuant RT ECL Imager (GE Healthcare, Little Chalfont, UK).

### Chemotaxis assay

Chemotaxis assay was performed using 96-well ChemoTx Chamber (Neuroprobe, Gaithersburg, MD) as described previously (17). Cells that migrated into the lower wells were lysed with 0.1% Triton X-100 and quantified using PicoGreen dsDNA reagent (Thermo Fisher Scientific).

### Calcium mobilization

The procedure has been described previously (18). Briefly, cells were loaded with 3 μM fura 2-AM fluorescence dye (Thermo Fisher Scientific). After washing, cells were placed on F3000 Fluorescence Spectrophotometer (Hitachi, Tokyo, Japan) and stimulated with recombinant chemokines. Emission fluorescence at 510 nm was measured upon excitation at 340 and 380 nm, and the fluorescence intensity ratio (R340/380) was obtained.

### DC mobilization and activation in mice

OVA (grade VI) and polyinosinic-polycytidylic acid (poly(I:C)) were purchased from Sigma-Aldrich (St. Louis, MO). C57BL/6 mice were injected intradermally in the right flank with OVA (100 μg) alone, OVA (100 μg) + mXCL1-WT (30 μg), OVA (100 μg) + mXCL1-V21C/A59C (30 μg), or OVA (100 μg) + poly(I:C) (10 μg). At indicated time points after injection, single cells were prepared from the skin and right inguinal lymph nodes, suspended in ice-cold PBS containing 0.1% bovine serum albumin (BSA) and 0.05% sodium azide (the staining buffer), and incubated with anti-mouse CD16/32 for 20 min to block the Fc receptors. After washing, cells were stained with anti-mouse CD45 (clone 30-F11), anti-mouse CD11c (clone N418), anti-mouse IA/IE (clone M5/114.15.2), anti-mouse CD19 (clone 6D5), anti-mouse CD103 (clone 2E7), anti-mouse CD11b (clone M1/70), anti-mouse XCR1 (clone ZET), anti-mouse CD40 (clone 3/23), anti-mouse CD86 (clone GL-1), and anti-mouse CCR7 (clone G043H7). Cells were analyzed on a FACSForttessa (BD Biosciences) or a FACSAria (BD Biosciences) using the FlowJo software (Tree Star Inc., Ashland, OR). All antibodies were purchased from BioLegend (San Diego, CA).

### Antigen uptake by CD103^+^ DCs

Alexa Fluor 488-conjugated OVA was purchased from Thermo Fisher Scientific. C57BL/6 mice were injected intradermally with Alexa Fluor 488-conjugated OVA (100 μg) + mXCL1-V21C/A59C (30 μg). After 6 h, cells were isolated from the skin samples, suspended in the staining buffer, and treated with anti-mouse CD16/32 for 20 min to block the Fc receptors. After washing, cells were stained with anti-mouse CD45, anti-mouse IA/IE, anti-mouse CD103 and anti-mouse CD11b as described above. Antigen uptake was analyzed by flow cytometry.

### CD8^+^ T cell responses

C57BL/6 mice were intradermally injected with OVA alone (100 μg), OVA (100 μg) + mXCL1-WT (30 μg), OVA (100 μg) + mXCL1-V21C/A59C (30 μg), or OVA (100 μg) + poly(I:C) (10 μg) three times at 1-week intervals. At 7 days or 4 weeks after the last immunization, cells were isolated from the draining lymph nodes and spleen. Cells were stimulated with MHC class I-restricted OVA-epitope (SIINFKEL) for 24 h. IFNγ-secreting CD8^+^ T cells were enumerated by flow cytometry. For OVA tetramer assay, isolated cells were incubated for 30 min with H-2Kb OVA tetramer-SIINFEKL-PE (MBL, Nagoya, Japan) and incubated with anti-mouse CD8 (clone KT15, MBL). After washing, cells were immediately analyzed by flow cytometry.

### *In vivo* T cell proliferation assay

C57BL/6 mice were intravenously injected with naïve OT-I spleen cells labeled with 5 mM CFSE (10^6^ cells for each mouse). After 24 h, mice were intradermally injected with PBS alone, OVA (100 μg) alone, OVA (100 μg) + mXCL1-WT (30 μg), OVA (100 μg) + mXCL1-V21C/A59C (30 μg), or OVA (100 μg) + poly(I:C) (10 μg). After 3 days, spleen cells were isolated and stained with anti-mouse CD90.1 and anti-mouse CD8. Proliferation of OT-I-derived CD8^+^ T cells was determined by flow cytometry using the CFSE dilution assay (8).

### *In vivo* CTL assay

Spleen cells were prepared from naïve C57BL/6 mice and incubated for 30 min at 37°C with OVA_257−264_ peptide in RPMI1640 medium containing 10% FBS and 50 μM 2-ME. The OVA_257−264_-plused spleen cells were then labeled with CFSE by incubation with 5-μM CFSE in PBS (CFSE^high^) for 10 min at 37°C. Naïve spleen cells were also labeled with CFSE by incubation with 0.5-μM CFSE in PBS (CFSE^low^ cells) for 10 min at 37°C. A mixture of 5 × 10^6^ CFSE^high^ cells and 5 × 10^6^ CFSE^low^ cells was intravenously injected into mice that were intradermally immunized with PBS alone, OVA (100 μg) alone, OVA (100 μg) + mXCL1-WT (30 μg), OVA (100 μg) + mXCL1-V21C/A59C (30 μg), or OVA (100 μg) + poly(I:C) (10 μg) three times at 1-week intervals. At 16 h after the last immunization, spleen cells were prepared from mice and analyzed for CFSE-labeled cells by flow cytometry. Specific lysis was calculated using the following formula: specific lysis (%) = 100–([CFSE^high^ immunized/CFSE^low^ immunized]/[CFSE^high^ control/CFSE^low^ control]) × 100.

### Antibody measurement

Serum samples were obtained from mice at indicated time-points. OVA-specific IgG titers were determined by ELISA following previously described protocols (19). End-point titers of OVA-specific antibody were expressed as the reciprocal log_2_ of the last dilution that showed more than 0.1 absorbance unit above the background.

### Tumor model

In a prophylactic model, C57BL/6 mice were intradermally immunized with OVA alone (100 μg), OVA (100 μg) + mXCL1-WT (30 μg), OVA (100 μg) + mXCL1-V21C/A59C (30 μg), or OVA (100 μg) + poly(I:C) (10 μg) three times at one-week intervals. Seven days after the last immunization, mice were intradermally inoculated with 1 × 10^6^ E.G7-OVA cells in the abdomen. In a therapeutic model, C57BL6 mice were intradermally inoculated with 1 × 10^6^ E.G7-OVA cells in the abdomen. After 7 days when tumors developed with a diameter of 5 to 6 mm, mice were intradermally immunized with OVA alone (100 μg), OVA (100 μg) + mXCL1-WT (30 μg), or OVA (100 μg) + mXCL1-V21C/A59C (30 μg) three times at 1-week intervals. To evaluate memory CD8^+^ T cell responses, C57BL/6 mice were intradermally immunized with OVA alone (100 μg), OVA (100 μg) + mXCL1-WT (30 μg), OVA (100 μg) + mXCL1-V21C/A59C (30 μg), or OVA (100 μg) + poly(I:C) (10 μg) three times at one-week intervals. Four weeks after the last immunization, mice were intradermally inoculated with 1 × 10^6^ E.G7-OVA cells in the abdomen. Tumor growth was monitored by measuring the major and minor axes using microcalipers. Tumor volume was calculated by the following formula: tumor volume (mm^3^) = (major axis (mm) × minor axis (mm))^2^ × 0.5236. Mice were euthanized when one of the two measurements was >20 mm.

### Statistical analysis

The Student *t*-test was used to analyze differences between two groups. One-way analysis of variance with the Tukey *post hoc* test was used for multiple groups. We considered *P* < 0.05 to be statistically significant.

## Results

### Generation of a structurally stable form of murine XCL1 termed mXCL1-VC21/A59C

**Figure 1:**
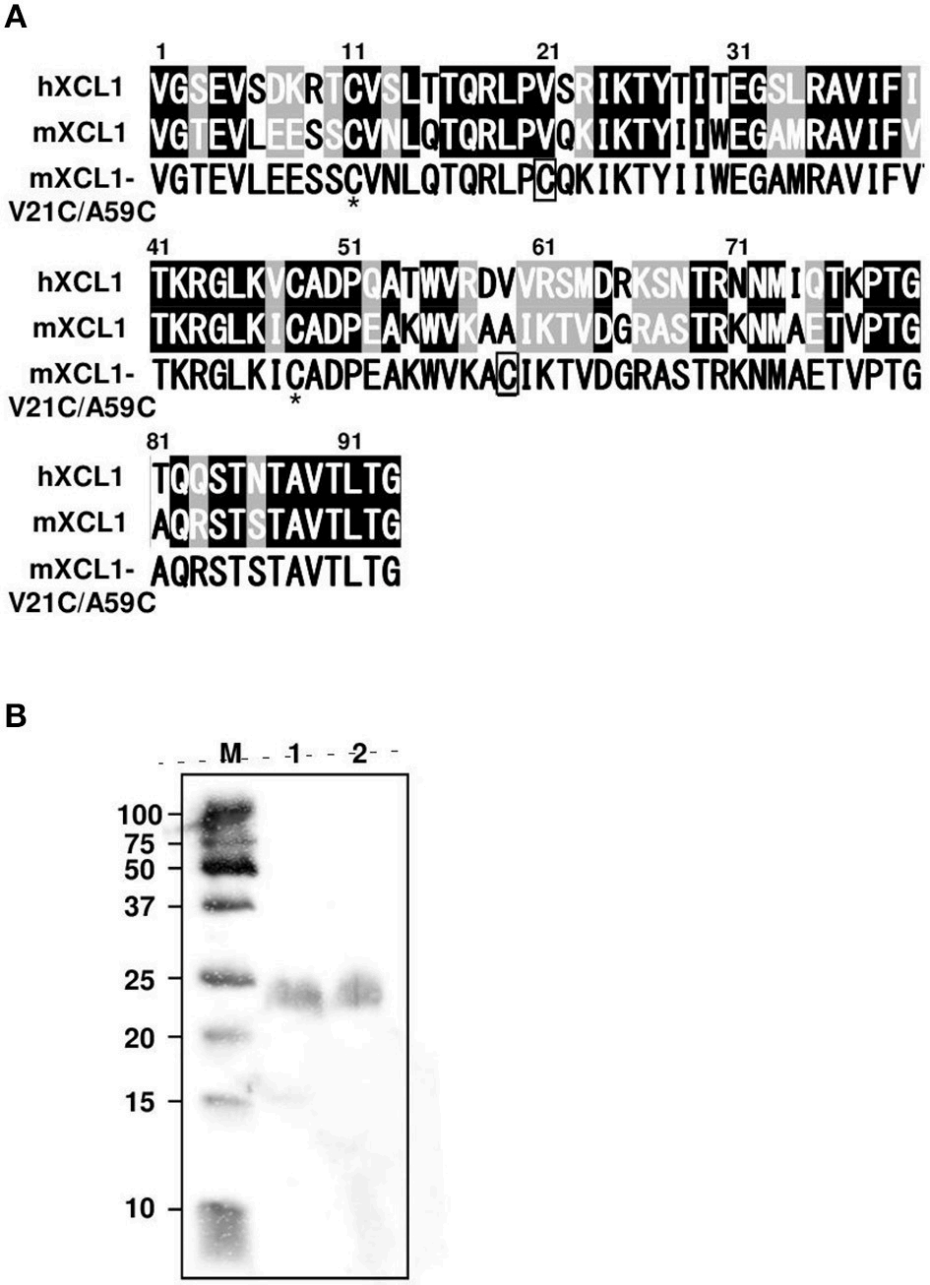
Generation of mXCL1-WT and mXCL1-V21C/V59C. **(A)** The mature amino acid sequences of hXCL1, mXCL1, and mXCL1-V21C/A59C. Identical amino acid residues are shown white on black, and similar amino acid residues are white on shade in the alignment of hXCL1 and mXCL1. The asterisk shows the conserved cysteine residues, while introduced cysteine residues are boxed in the amino acid sequence of mXCL1-V21C/A59C. **(B)** Western blotting of purified chemokines. Lane M: molecular marker. Lane 1: mXCL1-WT. Lane 2: mXCL1-V21C/A59C. Each purified sample was loaded at 100 ng protein/lane. After transfer, recombinant proteins were visualized by peroxidase-conjugated anti-His tag.

XCL1 lacks one of the two disulfide bonds that are commonly conserved in all other chemokines, resulting in an unstable structure (11). The addition of a second disulfide bond has been shown to stabilize the human XCL1 conformation and to enhance its chemotactic activity (12, 13). Since the amino acid sequences of human and murine XCL1 were highly conserved, exhibiting 61.3% identity and 84.9% similarity, we were able to construct the cDNA for murine XCL1 that contained the second disulfide bond (mXCL1-V21C/A59C) (Figure 1A). To generate the recombinant mXCL1-WT and mXCL1-VC21/A59C, 293-F cells were transfected with the respective expression vectors, pcDNA3.1-mXCL1-WT and pcDNA3.1-mXCL1-V21C/A59C, and the recombinant proteins were purified by His-tag column. The immunoblot analysis using the anti-His-Tag antibody detected purified mXCL1-WT and mXCL1-VC21/A59C both at about 24 kDa (Figure 1B). Although the amino acid molecular weight of XCL1 is 10 kDa, it is highly O-glycosylated and migrates at a much higher molecular weight on SDS-PAGE (20). Indeed, by HILIC HPLC, we confirmed that the purified mXCL1-WT and mXCL1-VC21/A59C were highly glycosylated and had only GalNAc, one of O-glycoside bonds (data not shown). There were thus no significant differences in the glycosylation profile of the two recombinant proteins. We also confirmed that endotoxin levels of purified recombinant proteins were < 0.1 EU/mg (data not shown).

### *In vitro* comparison of mXCL1-WT and mXCL1-V21C/A59C

**Figure 2:**
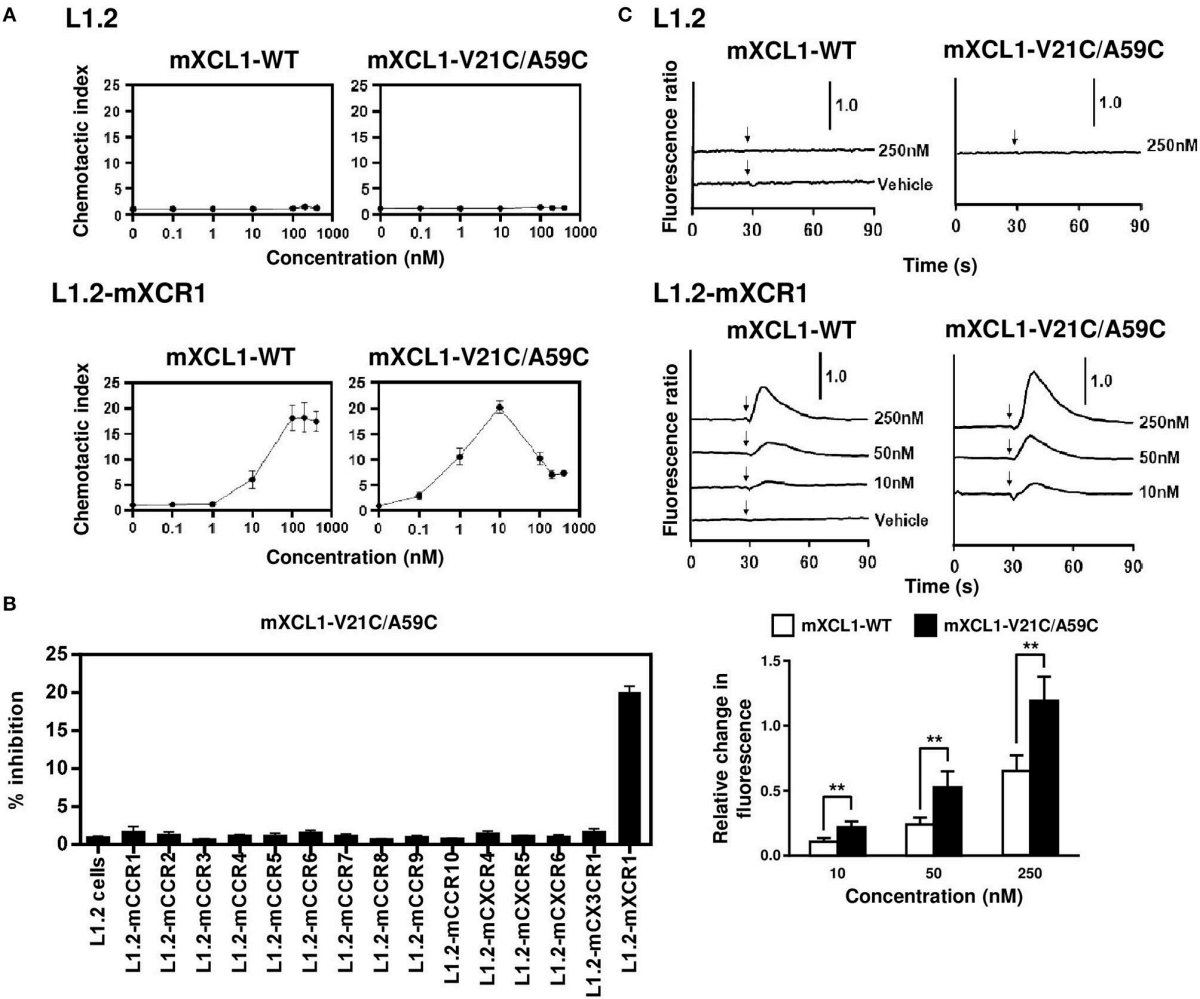
Chemotaxis and calcium mobilization assays. **(A)** L1.2 cells and L1.2 cells stably expressing murine XCR1 (L1.2-mXCR1) were used for cell migration to the indicated concentrations of mXCL1-WT or mXCL1-V21C/A59C. **(B)** L1.2 cells stably expressing indicated mouse chemokine receptors were used for cell migration to 10 nM mXCL1-WT or mXCL1-V21C/A59C. **(C)** L1.2 cells and L1.2-mXCR1 were loaded with fura 2-AM and stimulated with mXCL1-WT or mXCL1-V21C/A59C at indicated concentrations. Intracellular calcium mobilization was measured on a fluorescence spectrophotometer. Representative results from three separate experiments are shown. **P < 0.01.

We first evaluated the ability of mXCL1-WT and mXCL1-V21C/A59C to induce chemotaxis using murine L1.2 cells stably expressing murine XCR1 (L1.2-mXCR1). As shown in Figure 2A, both mXCL1-WT and mXCL1-V21C/A59C efficiently induced cell migration in L1.2-mXCR1, but not in control L1.2 cells, with a typical bell-shaped dose-response curve common to all chemokines and with a peak response at 100–200 nM and at 10 nM, respectively. Thus, mXCL1-V21C/A59C was about 10-fold more potent than mXCL1-WT. We also confirmed the receptor specificity of mXCL1-V21C/A59C. As shown in Figure 2B, mXCL1-V21C/A59C induced cell migration only in L1.2-mXCR1, but not in L1.2 cells stably expressing any other chemokine receptors. We next performed the calcium mobilization assay. As shown in Figure 2C, mXCL1-WT and mXCL1-V21C/A59C induced calcium mobilization in L1.2-mXCR1, but not in control L1.2 cells. Again, mXCL1-V21C/A59C was more potent than mXCL1-WT. Collectively, mXCL1-V21C/A59C was a highly potent agonist for mXCR1 compared with mXCL1-WT. The results were quite similar to those obtained with human XCL1-V21C/V59C reported in previous studies (12, 13).

### mXCL1-V21C/A59C efficiently induces accumulation of CD103^+^ cross-presenting dcs at the skin injection site

**Figure 3:**
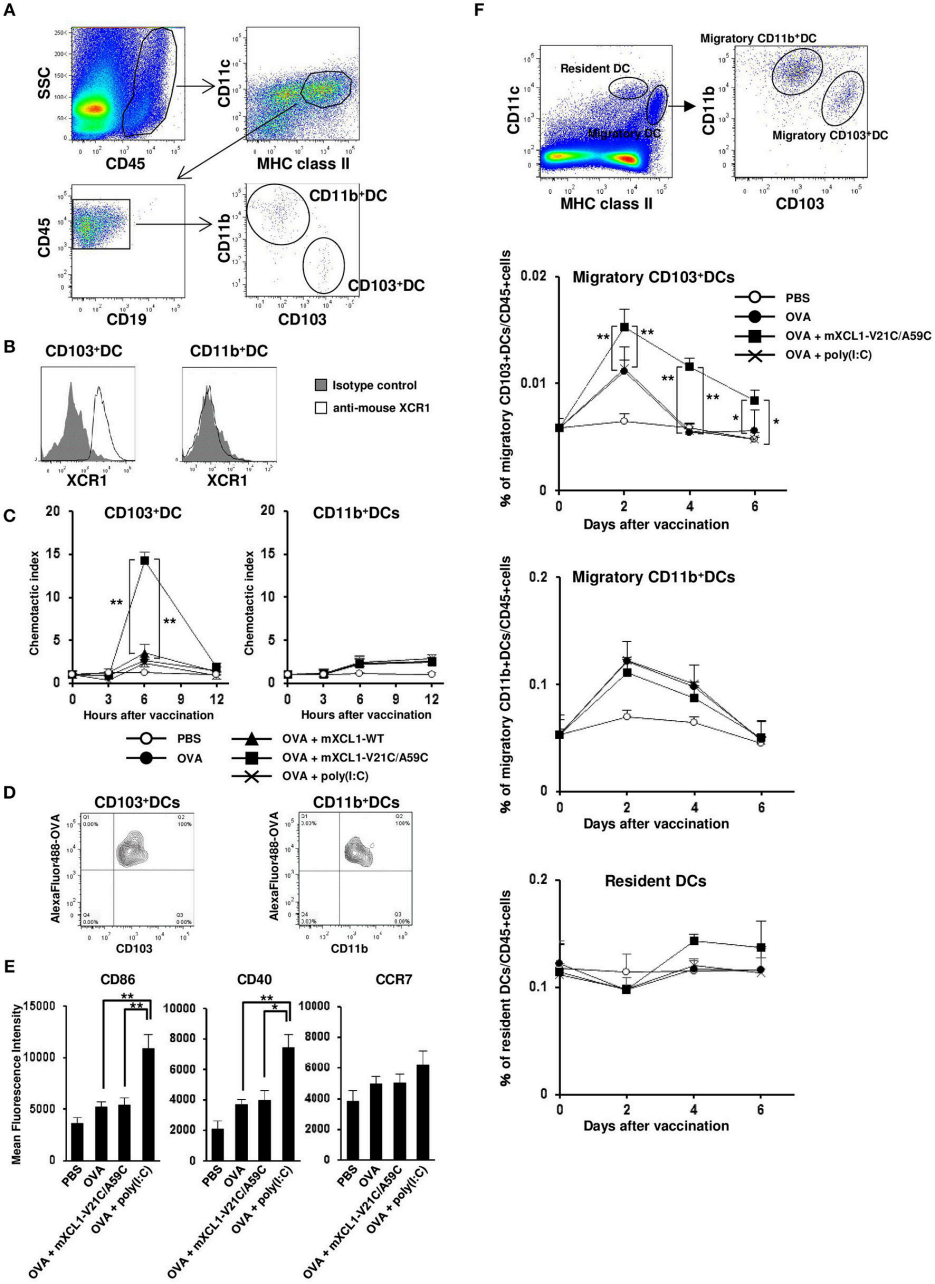
*In vivo* migration of CD103^+^ DCs by mXCL1-V21C/A59C. C57BL/6 mice were intradermally injected with OVA alone, OVA + mXCL1-WT, OVA + mXCL1-V21C/A59C, or OVA + poly(I:C). At indicated time points, skin cells were isolated from the injection sites and stained with anti-mouse CD45, anti-mouse IA/IE, anti-mouse CD11c, anti-mouse CD19, anti-mouse CD103, anti-mouse CD11b, and anti-mouse XCR1. DC numbers were quantified by flow cytometry. **(A)** Gating scheme of CD103^+^CD11b^−^ DCs and CD103^−^CD11b^+^ DCs in the skin by flow cytometry. **(B)** Expression of XCR1 on CD103^+^CD11b^−^ DCs and CD103^−^CD11b^+^ DCs. Representative results from at least 3 independent experiments are shown. **(C)** The accumulation of CD103^+^CD11b^−^ DCs (left) and CD103^−^CD11b^+^ DCs (right) in the injection sites was analyzed by flow cytometry. Chemotactic index = migrated cell numbers after vaccination/cell numbers before vaccination. The data are expressed as mean ± SE of results from 7 mice. **(D)** Antigen uptake by CD103^+^CD11b^−^ DCs (left) and CD103^−^CD11b^+^ DCs (right) in the skin. C57BL/6 mice were intradermally injected with Alexa Fluor 488-conjugated OVA + mXCL1-V21C/A59C. Six hours later, skin cells at the injection sites were isolated and stained with anti-mouse CD45, anti-mouse IA/IE, anti-mouse CD103 and anti-mouse CD11b. Antigen uptake was analyzed by flow cytometry. Representative results from at least 3 independent experiments are shown. **(E)** The surface expression of CD86, CD40, and CCR7 on CD103^+^CD11b^−^ DCs at 12 h after injection was analyzed by flow cytometry. The data are expressed as mean ± SE of results from 5 mice. **(F)** Gating scheme of migratory (MHC class II^high^ CD11c^int^) CD103^+^CD11b^−^ DCs, migratory CD103^−^CD11b^+^ DCs, and resident (MHC class II^int^ CD11c^high^) DCs in the draining lymph nodes by flow cytometry. The percentages of migratory CD103^+^CD11b^−^ DCs, migratory CD103^−^CD11b^+^ DCs, and resident DCs in the draining lymph nodes were quantitated by flow cytometry. The data are expressed as mean ± SE of results from 5 mice. *P < 0.05 and **P < 0.01.

mXCR1 is shown to be selectively expressed by CD103^+^CD11b^−^ DCs in mice and mediate their migration to mXCL1 *in vitro* (3). We confirmed that mXCR1 was expressed on CD103^+^CD11b^−^ DCs, but not on CD103^−^CD11b^+^ DCs, in the mouse skin (Figures 3A,B). We next compared the ability of mXCL1-WT and mXCL1-V21C/A59C to induce the accumulation of mXCR1^+^CD103^+^ DCs to the skin injection site. As shown in Figure 3C, intradermal injection of mXCL1-V21C/A59C with OVA as a model antigen significantly increased the percentages of mXCR1^+^CD103^+^ DCs, but not of CD103^−^ DCs, in the injection sites compared with OVA alone. On the other hand, intradermal injection of OVA with mXCL1-WT or the toll-like receptor (TLR) ligand poly(I:C) used as a control CTL-inducing adjuvant did not induce a significant accumulation of XCR1^+^CD103^+^ DCs or CD103^−^ DCs in the injection sites. We also confirmed that mXCL1-V21C/A59C alone induced the accumulation of CD103^+^ DCs at similar levels as mXCL1-V21C/A59C + OVA (data not shown). To further determine whether the accumulated XCR1^+^CD103^+^ DCs efficiently phagocytosed antigens, we injected Alexa Fluor 488-conjugated OVA together with mXCL1-V21C/A59C. As shown in Figure 3D, mXCR1^+^CD103^+^ DCs indeed efficiently phagocytosed Alexa Fluor 488-conjugated OVA. Of note, CD103^−^ DCs also efficiently phagocytosed Alexa Fluor 488-conjugated OVA.

### mXCL1-V21C/A59C efficiently induces migration of CD103^+^ cross-presenting dcs to the draining lymph nodes

Antigen-loaded DCs are known to be activated and to upregulate the surface expression of the co-stimulatory molecules such as CD86 and CD40 as well as the lymphoid homing chemokine receptor CCR7 (21). Of note, poly(I:C) was also reported to induce the upregulation of these molecules (22). We therefore analyzed the surface expression of CD86, CD40, and CCR7 on XCR1^+^CD103^+^ DCs. The surface expression levels of CD40 and CD86 on XCR1^+^CD103^+^ DCs were weakly increased in the skin injected with OVA alone compared with PBS control. The expression levels were much more increased in the skin injected with OVA + poly(I:C) (Figure 3E). In addition, poly(I:C) also slightly increased the CCR7 expression on XCR1^+^CD103^+^ DCs, although migratory (MHC class II^high^CD11c^int^) XCR1^+^CD103^+^ DCs in the draining lymph nodes were not increased (Figures 3E,F). On the other hand, mXCL1-V21C/A59C did not increase the expression levels of CD40, CD86 or CCR7 on XCR1^+^CD103^+^ DCs, but significantly increased migratory XCR1^+^CD103^+^ DCs in the draining lymph nodes (Figures 3E,F). The increases in migratory XCR1^+^CD103^+^ DCs in the draining lymph nodes were seen even 6 days after the last immunization. In contrast, mXCL1-V21C/A59C did not significantly increase migratory CD11b^+^ DCs and resident (MHC class II^int^CD11c^high^) DCs in the draining lymph nodes (Figure 3F). These results demonstrated that mXCL1-V21C/A59C not only efficiently induced the accumulation of XCR1^+^CD103^+^ DCs in the injection site but also their migration to the draining lymph nodes, although not so potent as poly(I:C) in the activation of DCs.

### mXCL1-V21C/A59C is more effective than mXCL1-WT in inducing CD8^+^ T cell responses

**Figure 4:**
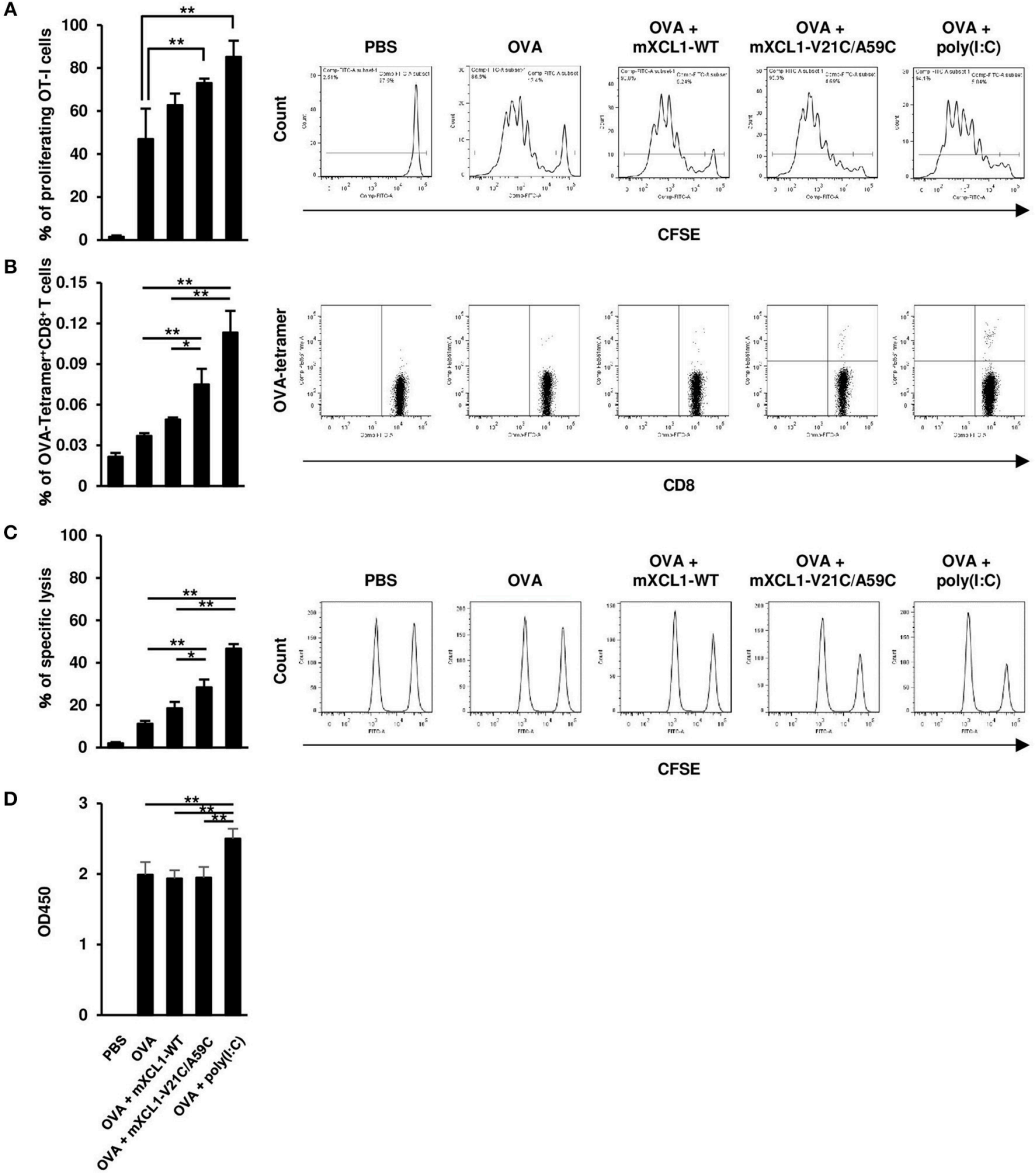
Effective induction of CD8^+^ T cell responses by mXCL1-V21C/A59C. **(A)** *In vivo* T cell proliferation assay. C57BL/6 mice were adoptively transferred with CFSE-labeled splenocytes from OT-I mice, and intradermally injected with PBS, OVA alone, OVA + mXCL1-WT, OVA + mXCL1-V21C/A59C or OVA + poly(I:C). After 3 days, splenocytes from these mice were stained with anti-mouse CD90.1 and anti-mouse CD8. Proliferation of OT-I-derived CD8^+^ T cells was determined by measuring the percentage of cells that have undergone at least one cell division. The data are expressed as mean ± SE of results from 4 mice. Representative results from all groups are shown on the right. **(B)** Tetramer assay. C57BL/6 mice were intradermally injected with PBS, OVA alone, OVA + mXCL1-WT, OVA + mXCL1-V21C/A59C or OVA + poly(I:C) three times with 1-week intervals. One week after the last injection, splenocytes from these mice were stained with anti-mouse CD8 and H-2Kb tetramer-PE loaded with MHC class I-restricted OVA-epitope (SIINFKEL). The data are expressed as mean ± SE of results from 4 mice. Representative results from all groups are shown on the right. **(C)** *In vivo* CTL assay. C57BL/6 mice were intradermally injected with PBS, OVA alone, OVA + mXCL1-WT, OVA + mXCL1-V21C/A59C or OVA + poly(I:C), three times with 1-week intervals. One week after the last injection, these mice were intravenously injected with OVA_257−264_-plused CFSE^high^ splenocytes (antigen-positive target), and nonpulsed CFSE^low^ splenocytes (negative control). After 16 h, CFSE-labeled target cells in the spleen were analyzed by flow cytometry. The data are expressed as mean ± SE of results from 4 mice. Representative results from all groups are shown on the right. **(D)** OVA-specific IgG production. C57BL/6 mice were intradermally injected with PBS, OVA alone, OVA + mXCL1-WT, OVA + mXCL1-V21C/A59C or OVA + poly(I:C), three times with 1-week intervals. One week after the last injection, serum samples were collected from mice and analyzed for OVA-specific IgG titer by ELISA. The data are expressed as mean ± SE of results from 4 mice. *P < 0.05 and **P < 0.01.

We next examined the effect of mXCL1-WT and mXCL1-V21C/A59C on the proliferation of antigen-specific CD8^+^ T cells *in vivo*. C57BL/6 mice were adoptively transferred with CFSE-labeled splenocytes from OT-I mice. After 24 h, mice were intradermally injected with OVA alone, OVA + mXCL1-WT, OVA + mXCL1-V21C/A59C or OVA + poly(I:C). After 3 days, proliferation of OT-I CD8^+^ T cells was determined. As shown in Figure 4A, OVA + mXCL1-V21C/A59C and OVA + poly(I:C), but not OVA + mXCL1-WT, significantly increased the percentages of proliferating CD8^+^ T cells compared with OVA alone. We also quantitated OVA-specific CD8^+^ T cells using a tetramer assay. As shown in Figure 4B, OVA + mXCL1-V21C/A59C and OVA + poly(I:C), but not OVA + mXCL1-WT, significantly increased the percentages of CD8^+^OVA-tetramer^+^ T cells compared with OVA alone.

We next assessed the OVA-specific CD8^+^ T cell activity 7 days after the last immunization using an *in vivo* CTL assay. C57BL/6 mice were transferred with CFSE-labeled and OVA_257−264_-plused splenocytes as target cells, and the killing of target cells was assessed by flow cytometry after 16 h. OVA + mXCL1-V21C/A59C and OVA + poly(I:C), but not OVA + mXCL1-WT, significantly enhanced the cytotoxic activity compared with OVA alone (Figure 4C).

We also tested the effect of the adjuvants on humoral immune responses. As shown in Figure 4D, only poly(I:C) slightly enhanced OVA-specific IgG production compared with OVA alone (Figure 4D). Of note, OVA alone induced a substantial IgG production possibly due to repeated immunizations with a high dose (100 μg).

### mXCL1-V21C/A59C induces antitumor effect in prophylactic and therapeutic tumor models

**Figure 5:**
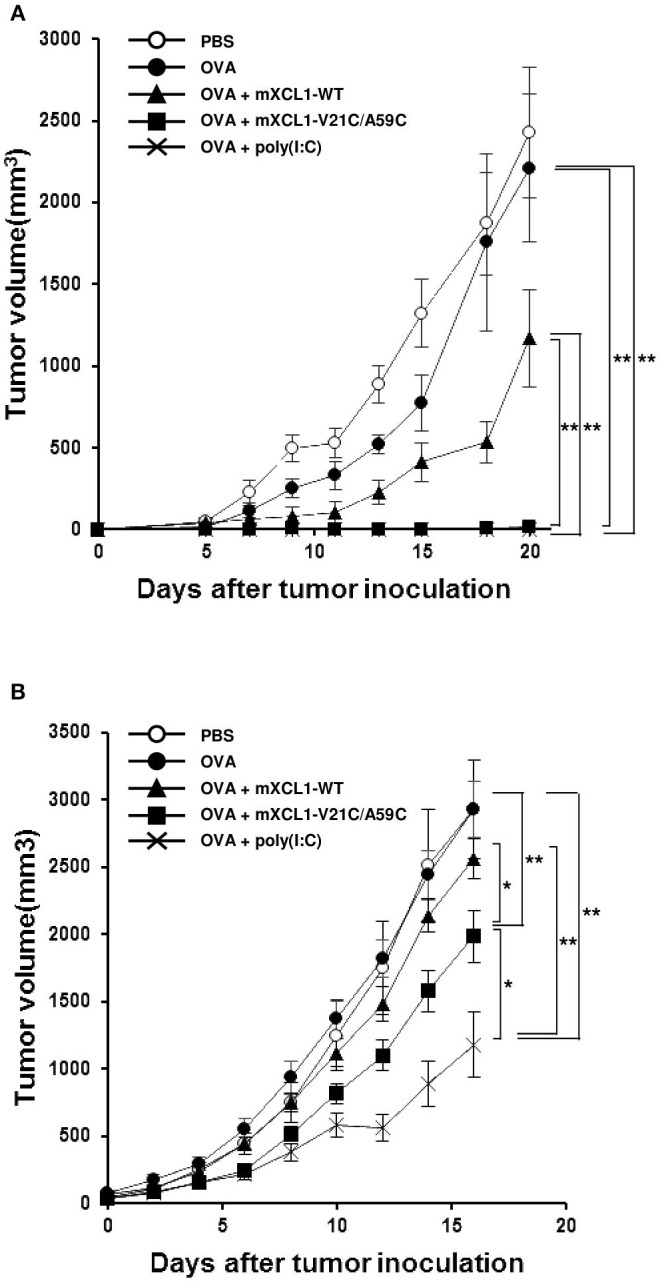
Antitumor activity in prophylactic and therapeutic tumor models. **(A)** Antitumor activity in the prophylactic tumor model. C57BL/6 mice were intradermally injected with PBS, OVA alone, OVA + mXCL1-WT, OVA + mXCL1-V21C/A59C or OVA + poly(I:C) three times with one-week intervals. One week after the last immunization, mice were challenged with E.G7-OVA cells in the flank. The tumor volume was calculated by measuring the major and minor axes of the tumor at indicated time points. Each point in the figure represents the mean ± S.E. from 8 mice. **(B)** Antitumor activity in the therapeutic tumor model. C57BL/6 mice bearing intradermal E.G7-OVA cells were injected with PBS, OVA alone, OVA + mXCL1-WT, OVA + mXCL1-V21C/A59C or OVA + poly(I:C) 3 times with 1-week intervals. The tumor volume was calculated by measuring the major and minor axes of the tumor at indicated time points. Each point in the figure represents the mean S.E. from 10 mice. *P < 0.05 and **P < 0.01.

We next examined the antitumor effect *in vivo* using E.G7-OVA cells that express OVA as a model tumor antigen. In a prophylactic model, C57BL/6 mice were intradermally injected with OVA alone, OVA + mXCL1-WT, OVA + mXCL1-V21C/A59C or OVA + poly(I:C), three times at one-week intervals. One week after the last immunization, E.G7-OVA cells were inoculated in the flank of mice and tumor growth was monitored. As shown in Figure 5A, tumor growth was weakly inhibited by immunization with OVA + mXCL1-WT compared with PBS or OVA alone. In contrast, tumor growth was almost completely inhibited by immunization with OVA + mXCL1-V21C/A59C or OVA + poly(I:C). In a therapeutic model, C57BL/6 mice were first inoculated with E.G7-OVA cells in the flank. After 7 days, mice were injected with OVA alone, OVA + mXCL1-WT, OVA + mXCL1-V21C/A59C or OVA + poly(I:C) three times at one-week intervals. As shown in Figure 5B, tumor growth was significantly inhibited by immunization with OVA + mXCL1-V21C/A59C or OVA + poly(I:C), but not with OVA + mXCL1-WT, compared with PBA or OVA alone. Of note, in consistence with the levels of induction of CD8^+^ T cell responses (Figure 4), poly(I:C) was more effective than mXCL1-V21C/A59C in the induction of antitumor effects.

### mXCL1-V21C/A59C induces memory CD8^+^ T cell responses

**Figure 6:**
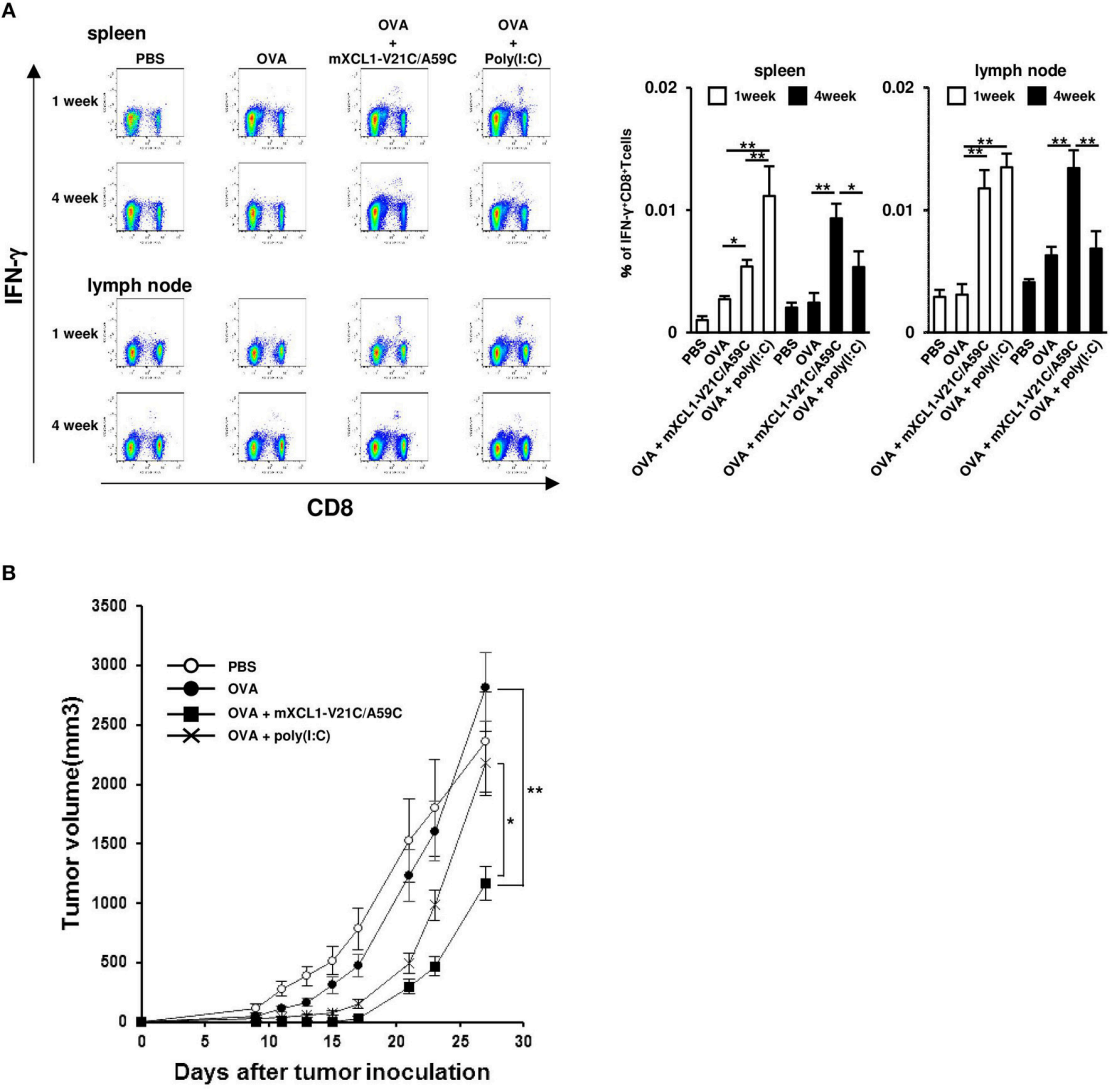
Induction of long-term memory CD8^+^ T cells. C57BL/6 mice were intradermally injected with PBS, OVA alone, OVA + mXCL1-WT, OVA + mXCL1-V21C/A59C or OVA + poly(I:C) three times with one-week intervals. **(A)** Measurement of IFN-γ^+^CD8^+^ T cells. One and four weeks after the last injection, cells were isolated from the spleen and draining lymph nodes. Isolated cells were incubated with MHC class I-restricted OVA-epitope (SIINFKEL) for 24 h and stained with anti-mouse IFN-γ, anti-mouse CD8, and anti-mouse CD44. The data are expressed as mean ± SE of results from 8 mice. Representative results from all groups are shown on the left. **(B)** Anti-tumor activity. Four weeks after the last injection, mice were challenged with E.G7-OVA cells in the flank. At indicated time points, tumor volume was calculated by measuring the major and minor axes of the tumor. Each point in the figure represents the mean ± S.E. from 7 mice. *P < 0.05 and **P < 0.01.

After immunization, antigen-specific effector CD8^+^ T cells expand with a peak at 7–10 days and then contract, resulting in a stable pool of antigen-specific memory CD8^+^ T cells by 4 weeks (23, 24). To evaluate memory CD8^+^ T cell responses, we measured OVA-specific IFN-γ-producing CD8^+^ T cells at 1 week (the effector phase) and 4 weeks (the memory phase) after the last injection. In the effector phase, mice immunized with OVA + mXCL1-V21C/A59C and those immunized with OVA + poly(I:C) had significantly higher frequencies of IFN-γ-producing CD8^+^ T cells in the spleen and lymph nodes than mice immunized with OVA alone (Figure 6A). In the memory phase, on the other hand, only mice immunized with OVA + mXCL1-V21C/A59C, but not those immunized with OVA + poly(I:C), had significantly higher frequencies of IFN-γ-producing CD8^+^ T cells in the spleen and lymph nodes than mice immunized with OVA alone (Figure 6A). We confirmed that IFN-γ-producing CD8^+^ T cells found in mice 4 weeks after the last immunization with OVA + mXCL1-V21C/A59C were positive for CD44, a memory marker, by flow cytometry (data not shown). We further examined the *in vivo* antitumor activity against E.G7-OVA at 4 weeks after the last immunization. As shown in Figure 6B, only mice immunized with OVA + mXCL1-V21C/A59C, but not those immunized with OVA + poly(I:C), significantly inhibited tumor growth in consistence with the levels of induction of OVA-specific memory CD8^+^ T cells (Figure 6A).

## Discussion

Previously, Tuinstra et al. generated a stable form of human XCL1 termed XCL1-V21C/A59C by introducing a second disulfide bond and demonstrated its enhanced chemotactic activity (12, 13). In the present study, we have generated a murine counterpart of this variant termed mXCL1-V21C/A59C (Figure 1) and tested its adjuvant activity for cross-presenting DCs. We confirmed that mXCL1-V21C/A59C was a much more potent ligand for mXCR1 than the wild-type mXCL1 (mXCL1-WT) in both chemotaxis and calcium mobilization assays (Figure 2). We have demonstrated that mXCL1-V21C/A59C is a highly effective adjuvant for cross-presentation and efficiently induced the accumulation of XCR1^+^CD103^+^ DCs in the injection site and their migration to draining lymph nodes, resulting in enhanced CD8^+^ T cell responses to OVA (Figures 3, 4). Thus, we were able to demonstrate that the tumor growth of OVA-expressing EL4 cells was strongly suppressed in mice immunized with OVA and mXCL1-V21C/A59C in both prophylactic and therapeutic settings (Figure 5). Furthermore, mice immunized with OVA and mXCL1-V21C/A59C acquired long-term memory CD8^+^ T cells to OVA (Figure 6).

TLR ligands such as poly(I:C) and CpG oligodeoxynucleotide are considered as highly promising adjuvants for the induction of CD8^+^ T cell responses. It has been demonstrated that TLR ligands activate DCs and enhance the production of inflammatory cytokines, including IL-12, resulting in the induction of Th1 cells and effector CD8^+^ T cells (25). However, IL-12 not only enhances the induction of effector CD8^+^ T cells, but also suppresses the induction of memory CD8^+^ T cells (25). In addition, although the lifespan of DCs can influence the induction of memory CD8^+^ T cells, it is known that highly activated DCs induce strong CD8^+^ T responses that in turn rapidly eliminate antigen-carrying DCs (26, 27). These observations may explain why poly(I:C) poorly induced memory CD8^+^ T cells to OVA compared with mXCL1-V21C/A59C (Figure 6). On the other hand, mXCL1-V21C/A59C not only induced the accumulation of XCR1^+^CD103^+^ DCs in the injection site and the draining lymph nodes but also maintained XCR1^+^CD103^+^ DCs in the draining lymph nodes for a prolonged period of time compared with poly(I:C) (Figure 3). Thus, the efficient induction of memory CD8^+^ T cells by mXCL1-V21C/A59C may be due to the efficient accumulation and maintenance of XCR1^+^CD103^+^ DCs in the draining lymph nodes. A stable form of XCL1 may thus provide a better alternative to TLR ligands as an effective CTL-inducing adjuvant.

Furthermore, not only the efficacy, but also the lack of strong adverse effects, is an important requirement for the clinical use of an adjuvant. In this context, while TLR ligands such as poly(I:C) and CpG oligodeoxynucleotide are highly efficient as an adjuvant for the induction of CD8^+^ T cell responses, these molecules are also known to induce various adverse effects such as fever, inflammation at the injection site, and tissue damage (22, 28). Thus, their strong adjuvant potency is also associated with strong adverse effects. On the other hand, the stable form of XCL1 may be useful for induction of CD8^+^ T cell responses without a wide-spread activation of immune cells. The administration of a high dose of mXCL1-V21C/A59C did not induce sustained activation of DCs in the draining lymph nodes (Figure 3). This may be consistent with the previous observations that XCL1-fusion vaccines did not activate DCs (8). Furthermore, XCL1 is an endogenous protein that is expressed at high levels in the spleen, thymus, intestine, and peripheral blood leukocytes, and at lower levels in the lung, prostate gland, and ovary (2, 5, 6). Thus, the administration of a stable form variant of XCL1 would cause little immunological responses by itself. Indeed, we found no local or general inflammatory responses in mice injected with mXCL1-V21C/A59C (data not shown). These results further support the notion that a stable form of XCL1 is a highly promising vaccine adjuvant for antigen cross presentation compared with other pro-inflammatory substances such as TLR agonists that activate a wide range of immune cells.

The intradermal injection of OVA and mXCL1-V21C/A59C did not significantly induce OVA-specific IgG responses in C57BL/6 mice (Figure 4). In contrast, it has been reported that the intradermal injection of DNA vaccines encoding XCL1-antigen fusion proteins by electroporation induces both CD8^+^ and CD4^+^ T cell responses in BALB/c mice (7). A similar observation has been made for XCL1-antigen fusion proteins delivered by laser-assisted intradermal delivery in C57BL/6 mice (9). The discrepancy may be due to the administration procedures used for these vaccines. DNA vaccination combined with electroporation or laser microporation is known to recruit various immune cells including DCs and to increase the expression of inflammatory genes, resulting in a broad range of T cell responses (9, 29). In this context, while cross-presenting DCs have only a poor ability to present antigens to CD4^+^ T cells via MHC-II, this ability is known to be enhanced by inflammatory cytokines and TLR ligands (30). Thus, the procedures used for DNA vaccines might have created an inflammatory milieu that favors a broad spectrum of antigen-specific T cell responses. The lack of humoral immune responses by the stable form of XCL1 may be quite advantageous for the selective induction of CD8^+^ T cell responses.

Previously, we have also developed transcutaneous immunization (TCI) systems, a hydrogel patch and a dissolving microneedle patch, both of which efficiently deliver antigens to DCs in the dermis (19, 31, 32). We have shown that these TCI systems efficiently induce antigen-specific CD4^+^ T cell responses (31, 33). Given that the stable form of XCL1 efficiently induces antigen-specific CD8^+^ T cell responses as shown in the present study, our TCI systems and the XCL1 adjuvant may provide us valuable tools singly or in combination to cover a broad spectrum of immunity through selective induction of CD4^+^ and CD8^+^ T cell responses.

In conclusion, we have demonstrated that a stable form of mXCL1 termed mXCL1-V21C/A59C induces the accumulation of cross-presenting DCs in the injection site and draining lymph nodes, providing an effective adjuvant activity for the induction of antigen-specific effector CD8^+^ T cells. Furthermore, we have demonstrated that mXCL1-V21C/A59C induces long-term antigen-specific memory CD8^+^ T cells. Thus, a stable form of XCL1 may be a highly promising adjuvant for the induction of effector and memory CD8^+^ T cells in the prevention and treatment of infectious diseases and cancer. A stable form of XCL1 may also be a better fusion partner for fusion vaccines in targeted delivery of antigens to cross-presenting XCR1^+^ DCs.

## Author contributions

KM, KK, FK, MK, YH, and ST performed experiments and made figures. OY and TN conceived and organized the study and analyzed data. KM, NO, AK, OY, and TN wrote manuscript.

### Conflict of interest statement

The authors declare that the research was conducted in the absence of any commercial or financial relationships that could be construed as a potential conflict of interest.
